# Characteristics and Risk Perceptions of Ghanaians Potentially Exposed to Bat-Borne Zoonoses through Bushmeat

**DOI:** 10.1007/s10393-014-0977-0

**Published:** 2014-09-30

**Authors:** Alexandra O. Kamins, J. Marcus Rowcliffe, Yaa Ntiamoa-Baidu, Andrew A. Cunningham, James L. N. Wood, Olivier Restif

**Affiliations:** 1 Disease Dynamics Unit, Department of Veterinary Medicine, University of Cambridge, Madingley Road, Cambridge, CB30ES England, UK; 2 Institute of Zoology, Zoological Society of London, Regent’s Park, London, NW1 4RY England, UK; 3 Center for African Wetlands, University of Ghana, P.O Box LG67, Legon, Accra, Ghana; 4 Department of Animal Biology and Conservation Science, University of Ghana, Legon, Accra, Ghana

**Keywords:** zoonoses, public health development, outbreak management, fruit bats, Ghana

## Abstract

Emerging zoonotic pathogens from wildlife pose increasing public health threats globally. Bats, in particular, host an array of zoonotic pathogens, yet there is little research on how bats and humans interact, how people perceive bats and their accompanying disease risk, or who is most at risk. *Eidolon helvum*, the largest and most abundant African fruit bat species, is widely hunted and eaten in Ghana and also carries potentially zoonotic pathogens. This combination raises concerns, as hunting and butchering bushmeat are common sources of zoonotic transmission. Through a combination of interviews with 577 Ghanaians across southern Ghana, we identified the characteristics of people involved in the bat-bushmeat trade and we explored their perceptions of risk. Bat hunting, selling and consumption are widely distributed across regional and ethnic lines, with hotspots in certain localities, while butchering is predominantly done by women and active hunters. Interviewees held little belief of disease risk from bats, saw no ecological value in fruit bats and associated the consumption of bats with specific tribes. These data can be used to inform disease and conservation management plans, drawing on social contexts and ensuring that local voices are heard within the larger global effort to study and mitigate outbreaks.

## Introduction

Over 300 distinct emerging disease events have been recorded in the last six decades, and the trend is accelerating (Jones et al. 2008). As the majority of emerging pathogens are zoonotic, originating largely from wildlife (Jones et al. 2008), increased human-wildlife contact is likely a major risk factor. Hunting, butchering and consumption of wild animals for food can potentially transmit zoonotic pathogens through animal bites, scratches, body fluids, tissues and excrement (Wolfe et al. 2005). The use of bats as food raises particular concern, as bats seem to host more zoonotic viruses per species than other taxa, including rodents (Luis et al. 2013).

Ghana hosts large colonies of *Eidolon helvum*, the straw-coloured fruit bat, and over 128,000 are hunted annually for food in the south of the country alone (Kamins et al. 2011). Recent studies are accumulating evidence that *E*. *helvum* may host several zoonotic pathogens including henipaviruses (Hayman et al. 2008), paramyxoviruses (Baker et al. 2012; Drexler et al. 2009), lyssaviruses (Wright et al. 2010) and Ebola virus (Hayman et al. 2010). Henipavirus spillovers from fruit bats to humans have occurred in Asia and Australia, where they caused fatal encephalitis and respiratory failure (Breed et al. 2006; Chua et al. 2000). Researchers have reported fatal spillover events of both lyssaviruses (Allworth et al. 1996) and filoviruses (Leroy et al. 2009) from bats to people in Africa. Although no spillover of henipaviruses to people have been reported in Africa, this may be due to a failure in identification as no systematic surveillance is undertaken.

From an ecological perspective, the susceptibility of fruit bats to overhunting (Harrison et al. 2011; Epstein 2009; Streubig et al. 2007) poses additional risks. *E*. *helvum*, currently listed as near-threatened (IUCN 2011), contributes to forest regeneration through seed dispersal and pollination of ecologically and commercially important species (Taylor et al. 2000). The loss or erosion of these ecological services could both harm the local environment and endanger the livelihoods of people dependent on these resources. Also, hunters, vendors and consumers who rely on *E. helvum* for secondary protein and income sources (Kamins et al. 2011) might start to experience a decline in quality of life if bat populations declined severely.

In the absence of conservation or public health management schemes related to bats, we had the opportunity to develop an evidence-based approach to help identify risks to the two sectors. Information on the people likely to be involved in bat-bushmeat activities would be important for the effectiveness of future interventions that may impact on *E. helvum* populations, such as restrictions on hunting or selling. In this study, therefore, we set out to investigate the sociological characteristics of communities who interact with bats and to assess their attitudes and perceptions regarding bats and disease.

There were three drivers behind this decision. First, in areas with limited resources, surveillance efforts must focus on groups most at risk. Such targeting requires information on the identities of those potentially at risk and on their beliefs and behaviours. We hypothesised that contact with bat bushmeat would cluster in particular subsets of the human population; for example, previous studies have demonstrated ethnic distinctions in preferences for bushmeat hunting and consumption (Willcox and Nambu 2007; Fa et al. 2002; East et al. 2005).

Secondly, while human health and rapid response to outbreaks are priorities, proceeding with an awareness of local perceptions about disease and intervention strategies can increase their effectiveness and minimise negative consequences (Dry and Leach 2010). Health organisations have sometimes reacted hastily to emerging zoonoses, discovering only later the complexities that accompany cross-species diseases. For example, in Gabon, the cultural inappropriateness of a top-down approach led to armed resistance against attempts to control the 2001 Ebola outbreak (Millerliri 2004). Also, communications about zoonotic disease risk can lead to a destructive backlash against wildlife populations—even without evidence supporting the efficacy of such actions. For example, in 2012, the tourism minister in Uganda planned a general cull of wildlife to stem an Ebola outbreak (The Africa Report 2012), without a clearly identified reservoir species for the virus or any assessment of the importance of targeted species for local livelihoods and ecosystems.

Finally, too often local community voices go unheard, despite representing those suffering at the epidemic coalface and often shouldering negative impacts arising from intervention measures. The identification of local actors most likely to be exposed to pathogens from bats provides an opportunity to protect them not only from infection but also from disregard for their needs in the management of an outbreak. Knowing how much people depend on bats for both survival and cultural purposes will help inform any restrictive policies needed, thus minimising negative reactions. For the welfare of both bats and humans, we considered also whether hunting pressures pose conservation concerns (reported in Kamins et al. 2011) along with public health risks.

To accomplish the three goals of (i) identifying target groups for surveillance, (ii) unearthing potential complexities relevant for outbreak management and (iii) understanding local actors and perceptions, we interviewed 577 Ghanaians across southern Ghana, including actors of the bat-bushmeat trade as well as members of the general public on markets. The data collected here expanded on our study of the structure of the Ghanaian bat-bushmeat trade (Kamins et al. 2011) to seek a broader understanding of the demographic and social characteristics of those involved. From these surveys, we developed and conducted a pilot study of a public education and livelihoods intervention as a method for managing disease risks from bat bushmeat. Data on perceptions and beliefs pertaining to bats gave us clues to the context in which such an intervention would operate. Finally, we compared people’s beliefs about patterns of bat usage with those actually reported to better understand how perceptions and realities might diverge.

## Methods

### Study Site

We conducted our surveys in two cities (Accra and Kumasi), a town (Nkawkaw) and surrounding villages and two rural areas (the southern part of the Volta Region and the Afram Plains). All were located within southern Ghana. Ethical approval was provided by the Zoological Society of London and the Noguchi Memorial Institute for Medical Research in Accra, Ghana.

1. Accra is the capital of Ghana, with an urban population of ca. 2.64 million (Ghana Statistical Service 2011).
2. With ca. 2.04 million inhabitants, Kumasi is the second largest city in Ghana, located northwest of Accra in the Ashanti region (Ghana Statistical Service 2011). In Kumasi there is a large central market, where bushmeat is sold, as well as a bushmeat-specific market (Atwemonom).
3. Nkawkaw is a small, rural town in the hills of the Eastern Region, located along the major road that runs from Accra to Kumasi.
4. The Volta Region stretches along the eastern border of southern Ghana. Each village in the Volta Region has a central market that is active on a given market day each week, which sell bushmeat when it is available.
5. The Afram Plains lie in the easternmost part of the Eastern region, on the western shores of Lake Volta. As in other parts of Africa (Milner-Gulland and Bennett 2003), farmers in the Volta Region and the Afram Plains often hunt bushmeat for food, for additional income and to protect their crops.

### Questionnaires

Fieldwork took place over five one-month long sessions from November 2009 to September 2011. There were three main parts to the data collection: a survey of bat-human interactions, interviews of broad population samples and targeted interviews of people engaged in bat-related activities.

We used three different questionnaires. The first two questionnaires, described by Kamins et al. (2011), covered socio-demographic information of the respondent, interactions with bats, beliefs about bat bushmeat, perceptions of disease risks from bats and general meat preferences (see Appendix 1). As described in Kamins et al. (2011), we initially directed interviews to hunters we witnessed shooting *E. helvum* and to vendors selling *E. helvum* in the markets. These interviewees provided details of other hunters and vendors, and thus we were able to penetrate the entire commodity chain through a cascade effect. In addition, we conducted convenience sampling in each of our sites by standing at the entrance to each main market place, or along the only main road of each small village, in our study sites and by choosing for interview the first person who walked by at exactly 5 min after we had completed our previous interview. We asked any respondent who ate bats (identified as a consumer) or prepared bats for consumption (whether or not the respondent ate the bats him/herself, identified as a preparer) and then further questions about these activities, such as frequency or methods. Any respondent that sold (either as a hunter-seller or a commercial vendor) or hunted bats completed an additional questionnaire asking for details such as the frequency and locations of bat hunting or purchasing of bats; respondents that did both completed surveys for both activities. The basic counts from these surveys were analysed in Kamins et al. (2011) to examine sustainability of bat hunting in Ghana, as well as to provide details including location and method of bat hunting; here, we examine the more complicated socio-demographic information on these surveys.

Once we identified the dominant socio-demographics and perceptions of participants in these activities, we wanted to better understand how these data might relate to intervention possibilities and challenges. We therefore conducted additional interviews with eighteen selected vendors across the study area and with eight Volta hunters using a third questionnaire (See Appendix 2). These interviews began with a few pre-test questions on the environmental importance of bats and disease risk from bats, followed by questions relating to perceptions about bats, feelings on current and proposed bushmeat-management interventions and past and future plans for personal involvement with bat bushmeat. Interviewers then shared and discussed a brief education piece with verbal explanations of either the dangers of bat-borne diseases (Appendix 3) or important ecological roles of bats (Appendix 4), before concluding with the same pre-test questions. Respondents were randomly assigned identification numbers; all even numbers received one education piece and all odd numbers received the other. Because the two different interview/education groups received the same set of questions, they served as controls for one another.

### Analysis

We analysed the frequencies of consumption, preparation, hunting and selling of bats using a generalised linear model, with the following explanatory variables: gender, age, region, education, study site, participation in one or more of the bat-bushmeat activities, personal preference of meat type (fish, domestic, or bushmeat) and perception of any disease risk from bat-bushmeat activities or consumption. We used ancestral region only in our analysis, excluding ethnic group, because Ghana hosts many ethnic groups and we discovered the two variables correlate closely; Ghanaians commonly reported their ancestral region as that from which their tribe originates regardless of where the individual was actually raised. All the models obtained by including or excluding each explanatory variable were ranked according to Akaike’s Information Criterion (AIC). We selected the subset of models within two AIC units of the best model. We further examined the highlighted variables using proportion tests. We used R version 2.12.2 (R Development Core Team 2011) for all our statistical analyses.

## Results

In total, we interviewed 577 Ghanaians, 551 using the general survey and 26 in in-depth focus interviews (Tables 1, 2). Actual numbers of bats hunted and sold were analysed in Kamins et al. (2011). The average age of the all the respondents was 39.0 years, and 52% were men.

**Table 1:** Distribution of Respondents in the General Survey

	Respondent category				Sampling method		Total interviews
	Hunters	Vendors	Consumers	None	Convenience	Cascade	
Accra (Urban)	32 (15)	15 (12)	55 (42)	141	196	18	214
Afram (Rural)	23 (16)	0 (0)	38 (17)	26	60	6	66
Kumasi (Urban)	9 (3)	7 (4)	27 (16)	62	77	15	92
Nkawkaw (Rural)	5 (2)	8 (7)	31 (18)	42	72	9	81
Volta (Rural)	26 (22)	18 (14)	90 (42)	16	66	32	98
Total	95 (59)	48 (37)	241 (135)	287	471	80	551

Numbers in parentheses indicate number of the total that were actively participating in indicated activity, e.g. 15 (12) means of 15 vendors, 12 had sold bats within the previous 12 months. Respondents can occupy multiple categories, and therefore the overall total of the respondent types exceeds 551.

**Table 2:** Totals for Focus Group Respondents

Location	Hunters	Vendors	Total
Accra (Urban)	0	7	7
Volta Region (Rural)	7	12	19
Total	7	19	26

### Evaluation of Potential Risk of Bat-Bushmeat Behaviours

**Figure 1:**
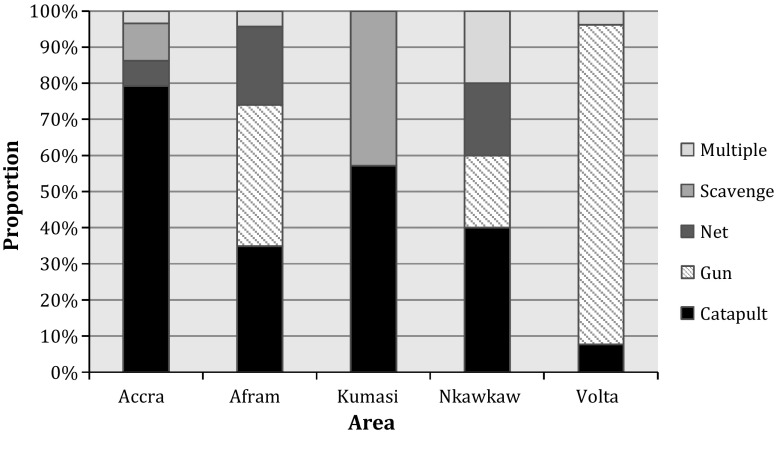
Different hunting methods across the interview sites. Accra and Kumasi are urban areas, while Afram, Nkawkaw and Volta are rural.

Hunters used shooting, netting, scavenging and catapulting in varying proportions across the study sites, with shooting occurring virtually only in the rural areas (Fig. 1). All hunters reported handling live bats, coming into contact with bat blood and getting scratched and three hunters mentioned being bitten. Scavenged bats were collected alive, usually when a branch broke and bats fell to the ground. Four interviewees explained how people would fight over the bats when a large branch fell, sometimes even lying down on top of bats to prevent others from taking them. In the scramble, people sustained bites and scratches. No hunter reported using protective measures, such as gloves.

Bats were never observed or reported being sold live or kept alive at the marketplaces, even in the single observation of bats being sold along the roadside [unlike common practices in Asia (Streubig et al. 2007; Harrison et al. 2011)]. Twenty-five percent of 48 vendors received fresh carcasses, while the rest bought only smoked bat meat. Of 132 people who prepared bats, 37% gutted the bat before roasting or smoking it; 2% of preparers reported adding fresh bat intestines into soup. Sixty-nine per cent made soup from the bats, while 8% roasted them as kebabs. Two-thirds smoked the bats before using them to prepare food. Consumers (*n* = 241) received bats fresh (44%), smoked (32%), a combination (8%), or as prepared food (16%). There were no reports of eating raw bats. Several respondents mentioned the belief that stirring milk with a bat’s head on a stick gave good luck and improved the milk froth.

### Characteristics of Actors

The multi-model inference approach highlighted a few key factors affecting the probability of hunting, preparing, selling or eating bats, these were: participation in other bushmeat activities, interview area, age, gender and education (Table 3). Several models obtained very similar AIC scores, leaving some uncertainty on the importance of those factors. There was moderate evidence for meat-type preference but only weak evidence for ancestral region and perception of disease. In the following sections we look more closely at each variable.

**Table 3:** Comparison of Top Models with ΔAIC < 2 for the Four Response Variables, Ranked on AIC

Variable	Age	Area	Ed.	Gender	Pref.	Region	Other	Risk	AIC weight
Hunt	X			X			X		0.24
	X		X	X			X		0.23
	X			X	X		X		0.18
	X		X	X	X		X		0.16
	X		X	X			X	X	0.10
	X			X			X	X	0.09
Sell		X	X		X		X		0.23
		X	X		X		X	X	0.17
	X	X	X		X		X		0.15
		X	X	X	X		X		0.12
	X	X	X		X		X	X	0.12
		X	X	X	X		X	X	0.11
		X	X				X		0.10
Prepare	X	X	X	X			X		0.31
	X	X		X			X		0.24
	X	X	X	X	X		X		0.17
		X	X	X			X		0.14
	X	X					X		0.13
Eat	X	X	X	X		X	X	X	0.51
	X	X	X	X		X	X		0.49

“Area” refers to the location of the interview (in one of the five study areas), “Ed.” indicates the variable of education level, “Pref.” is respondent preference for bushmeat, domestic meat or fish, “Region” is the home region of the respondent, “Other” is whether the respondent participates in other bat-bushmeat activities than the one being tested, and “Risk” refers to whether the respondent perceived a risk of disease from any of the activities.

### Interview Area and Ancestral Region

**Figure 2:**
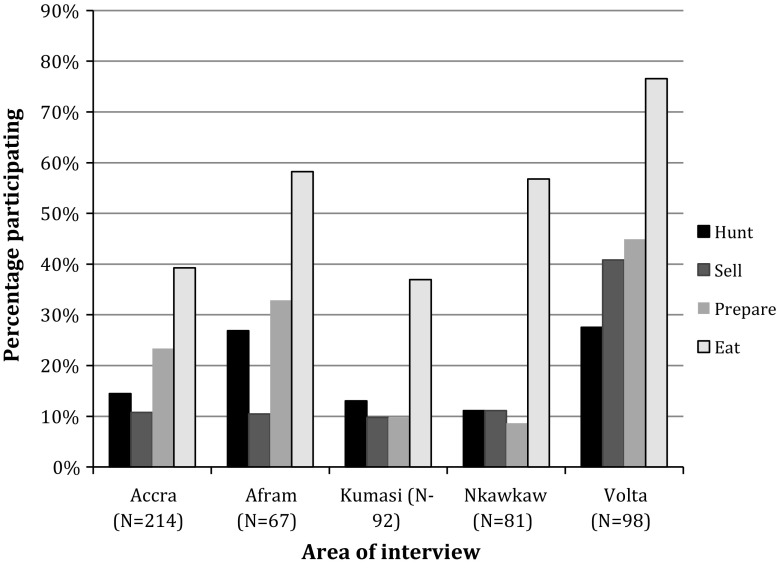
Proportions of respondents participating in different bat-bushmeat activities, by interview area. Accra and Kumasi are urban areas, while Afram, Nkawkaw and Volta are rural. Respondents can be included in more than one category (therefore totals will be >100%).

**Figure 3:**
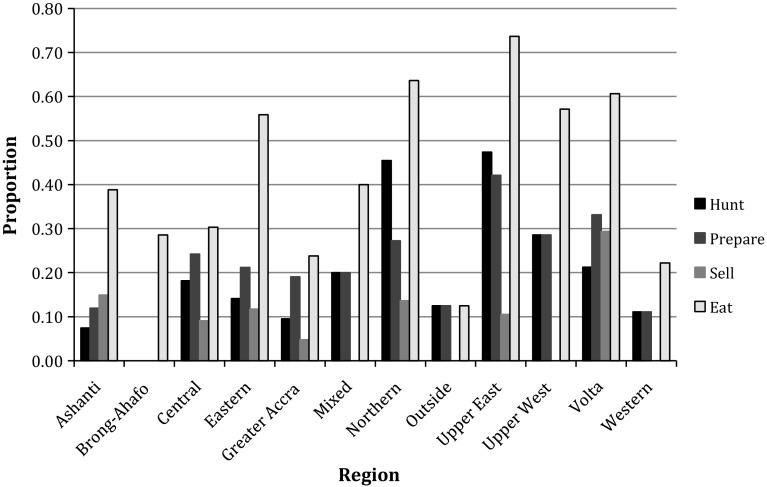
Proportions of respondents from different ancestral regions who participated in the various bat-bushmeat activities.

Participation in bat-bushmeat activities varied across the interview areas (Fig. 2). Although participation varied across ancestral regions (Fig. 3), the overall effect was weak, possibly due to interaction with interview area for some groups. For example, among respondents ancestrally from Volta who are currently living there, the proportion reporting bat consumption was 78% (*N* = 96), compared to 27% (*N* = 58) for those living in Accra (Chi squared test, *P* < 0.0001). Respondent in the Volta region also had the highest proportions participating in hunting, butchering and selling. For regularity of engagement in bat trade, please see Kamins et al. (2011).

### Age

**Figure 4:**
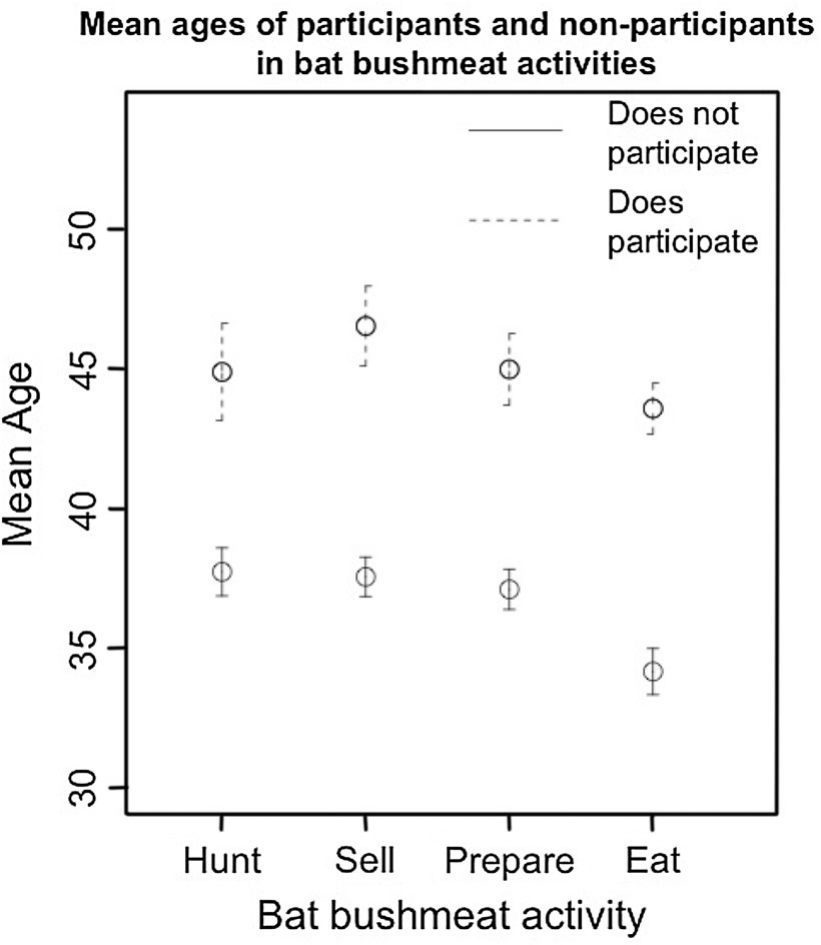
95% confidence intervals for the mean age of people who do and do not participate in the various bat-bushmeat activities.

Participants in all four bat-bushmeat activities were significantly older, on average by seven to 10 years, than non-participants (Fig. 4, *t* test, *P* < 0.0001, *N* = 551).

### Gender

All but two hunters were men; the two female hunters interviewed would scavenge fallen bats or help hit bats with sticks after hunters had shot them. While 17% of women interviewed took part in preparing bats, the proportion was 31% among all male respondents, but only 12% in non-hunting men. Sixty percent of men interviewed ate bats compared to 42% of women. Only one vendor was a man, who moved from town to town. All other male sellers were hunters who sold their bats either to market vendors or straight to customers.

### Education

**Figure 5:**
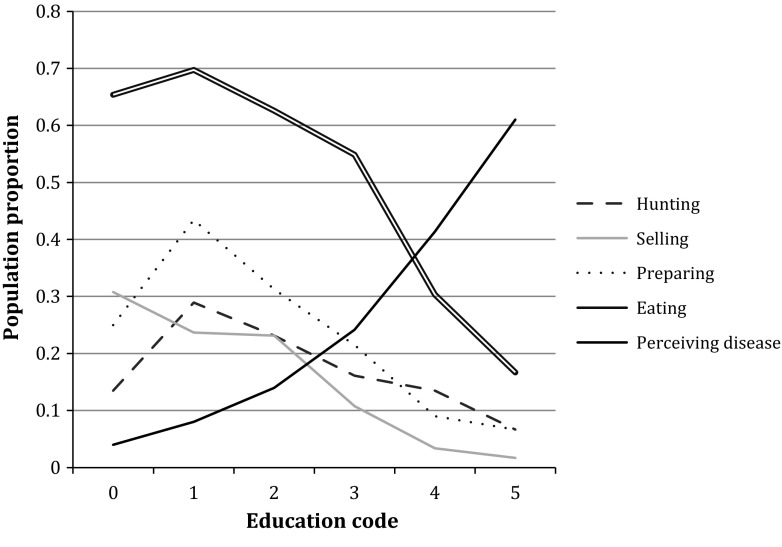
Participation in different bat-bushmeat activities and perception of disease risk from such participation based on education level. Education levels were coded as follows: *0* no formal schooling, *1* some primary education, up to form 4, *2* some secondary education, *3* secondary complete, *4* technical diploma or equivalent, *5* university education.

The more-highly educated a respondent, the less likely they were to hunt, sell, prepare, or eat bats (Fig. 5). Respondents from Accra and Kumasi had significantly higher levels of education than those from Volta, Nkawkaw and Afram (six-sample Chi squared test, *P* < 0.0001, *N* = 517).

### Perceptions

When asked who they thought ate bats, 50% of respondents (*N* = 551) indicated that bat consumption was due to culture, tradition, or tribe. Almost 25% indicated specifically the Kwahu tribe, by far the tribe that was most frequently cited as eating bats. Another quarter of the respondents felt that “all kinds of people eat bats.” Fewer than 4% considered wealth to be related to the consumption of bats, with 3% believing poor people eat bats and 1% that rich people eat bats.

When bat consumers were asked the open question of why they personally ate bat meat, the most common response (43%) was because it tasted good, while only 14% said it was because bat meat was a traditional food of their tribe. Other reasons included that bats were just another source of meat (20%), that bat meat provided health benefits (9%), curiosity (7%), or recommendation by friends (6%). One frequent health benefits mentioned was that, due to the bats’ diet, their meat is full of good vitamins and nutrients; a second benefit recorded was that bat meat was low in cholesterol. There were two reports in Nkawkaw about bats being eaten during funeral ceremonies. No other direct cultural uses for bats were cited. Two respondents mentioned they had stopped eating bat meat because it had become too expensive to purchase, and two vendors said bats had become too expensive now to buy as part of their inventory.

In meat-preference rankings, bat eaters chose grasscutter *Thryonomys* *swinderianus* (a large rodent commonly hunted and eaten in western Africa) as their favourite meat while non-bat consumers selected goat and chicken. Bat ranked third among consumers. The proportion of bat consumers was 55% among respondents who preferred bushmeat to domestic meat and only 32% among those who preferred domestic meat. Overall, 53% of respondents preferred bushmeat in general, while 13% stated no preference between domestic meat and bushmeat.

Overall, 23% of respondents perceived a disease risk from a bat-bushmeat activity, with significantly more respondents associating risk with bat consumption than bat preparation or hunting (Table 4, Chi squared test, *P* < 0.001). The perception of risk was lower in rural areas than in cities (Chi squared test, *P* < 0.0001): in the cities of Accra and Kumasi 37 and 31% of respondents perceived risk, respectively, compared to 12, 8 and 9% in the rural areas of Afram, Nkawkaw and the Volta Region, respectively. Higher education levels were related to an increase in the perception of disease risk (proportion trend test, *P* < 0.0001, *N* = 498, Fig. 4). There was a negative association between disease risk perception and participation in bat preparation (Fisher’s exact test, *P* = 0.035) or participation in bat consumption (Chi squared test, *P* < 0.0001, see Table 4).

**Table 4:** Proportions of Respondents to the Questions “Do You Think It Is Possible for Someone to Get Sick from Just Hunting/Butchering/Eating a Bat?” and Either Participated or Did Not Participate in the Corresponding Activity

	Hunt				Prepare				Eat				Any Participation	
	Risk	No Risk	DK	Total	Risk	No Risk	DK	Total	Risk	No Risk	DK	Total	Any Risk	No Risk
Participates	2%	86%	12%	91	5%	80%	15%	123	7%	73%	13%	285	9%	91%
Does not	6%	72%	22%	431	11%	70%	18%	402	22%	63%	23%	238	27%	73%
Total	5%	72%	20%	522	10%	72%	18%	525	14%	69%	17%	523	17%	83%

Those who replied, “only injured, not sick” were included in the “no” category (34 for hunting, 3 for butchering, 0 for eating). Non-responses were excluded. The “any participation” category gives the percentages of respondents who participated in at least one activity, or participated in none; “any risk” indicates whether they felt that for at least one activity, there was a disease risk. “No risk” indicates those who saw no risk of disease from any bat-bushmeat activity.

### Education Pilot and Focus Question Results

**Figure 6:**
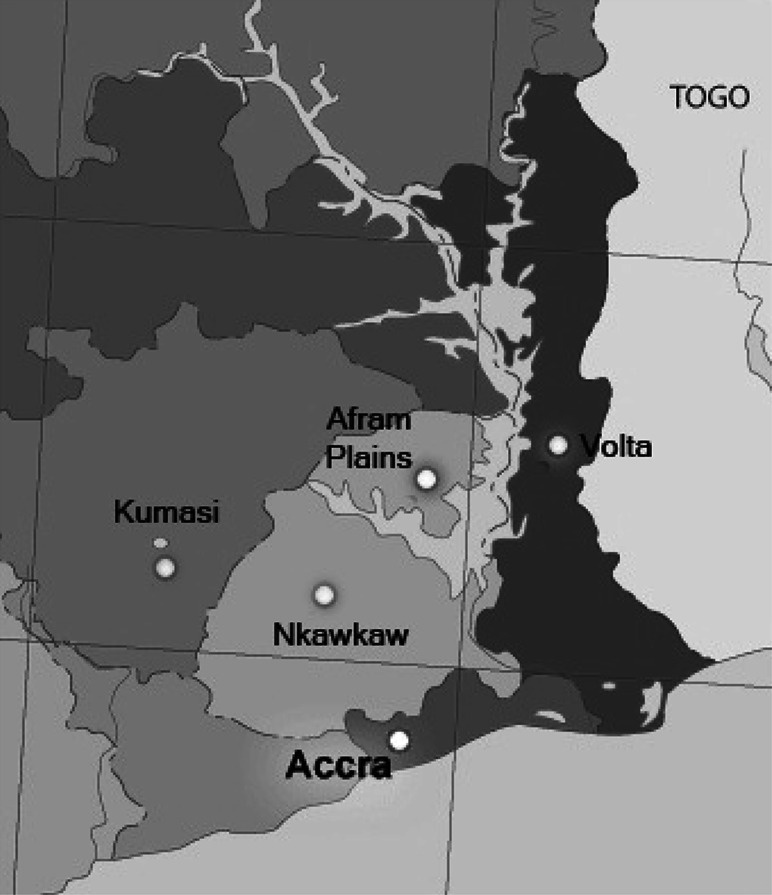
Map of study area.

Our brief education pieces substantially improved understanding of bat-borne disease risk and the environmental importance of fruit bats (Table 5, *N* = 23 respondents completed). However, when asked if this knowledge would impact respondents’ future decisions to hunt, butcher or sell bat bushmeat, only 55% of respondents indicated that they no longer wished to take part in these activities (Table 6, *N* = 26 respondents with responses for multiple activities). The most common response to the open question about what would make them not hunt, sell or butcher bats, was if they could get sick from the bats (8), followed by stopping if they found a better economic opportunity (6). Three said harsher laws or fines would stop them, while three said that a lack of demand or profit would; two more cited environmental impact as a reason to stop hunting, while two others said nothing would make them stop and two said they had largely stopped already (Fig. 6).

**Table 5:** Proportions of Respondents Whose Answers to the Question Changed From ‘no’ to ‘yes’, as Compared to Those Who Stayed at ‘no,’ After Undergoing a Brief Education Piece on Disease Risk From Bats or Environmental Value of Bats

	Disease		Environment	
	Education group	Control group	Education group	Control group
Changed answer from “No”	91%	33%	90%	10%
No change	9%	67%	10%	90%
Total	11	12	10	10

For each question, the control group comprised those who received the alternative education piece rather than the one relevant to the question. Respondents who answered “yes” to begin with were excluded from this comparison.

**Table 6:** Changes in Answer to the Question, “Will you hunt/butcher/sell bats in the future?” from Before and After Education Pieces About the Environmental Benefits of Bats and the Potential Disease Risks From Bats

Change	Hunt in future?			Butcher in future?			Sell in future?			Total
	Environment	Disease	Total	Environment	Disease	Total	Environment	Disease	Total	
Changed answer from “Yes”	2	3	5	5	1	6	4	7	11	22
No change	2	1	3	2	1	3	8	4	12	18
Total	4	4	8	7	2	9	12	11	23	40

Only respondents who started with “yes” to participating in that activity in the future are shown below. There were 26 respondents, with some giving multiple responses as they participated in multiple activities.

When asked if a 10 GHc fine (about US$7) would stop respondents from hunting or selling bats, 77% said yes; when increased to 100 GHc (about US$70), this proportion increased to 85% of the 26 respondents. The respondents who would continue said that they could make enough profit to cover the fine. Two said nothing could make them stop, and six were undecided. Three-quarters of the focus group members said they would definitely sell, buy, or raise farmed grasscutter and three more said they would consider it.

Only one of the focus respondents knew the dates for the closed season in Ghana, during which hunting for all animals except grasscutter is forbidden. Six were partially right (e.g., knew that it started sometime in the dry season), five were completely wrong and fourteen said they did not know or could not answer. The actual dates for the closed season are August 1 to December 1 (GNA 2012).

When asked in an open-ended question, before the education piece, what value bats have for people, only four responses were given: no value (14%), economic value (30%), meat (30%) and both meat and money (26%). Sixteen percent felt that people depended on bats for survival, 26% felt that people depended on the money they earned from selling bats and 58% felt that people would manage if bats disappeared.

## Discussion

Three overarching themes arise from our Ghana-based study, with some global implications. After previous work demonstrated that bat bushmeat was a common source of bat–human interactions in Ghana (Kamins et al. 2011), the results presented here show that understanding the characteristics of those involved can be useful for focussing the efforts of health protection programmes and provide clues to potential impediments to interventions.

The results of our questionnaires and focus groups showed that bat-bushmeat actors comprised a complex array of groups. The older age of participants versus non-participants could imply a number of scenarios. The first is that there is a decrease in youth interest in bat bushmeat. The second is that bats are easier for less active men to hunt than other bushmeat species. A third option is that selling and hunting bats require resources not available to younger people, such as funds for transport. Market women, for example, have to build up capital over years to be able to buy more expensive items to sell, meaning that older (and thus longer-operating) women may be able to purchase different sets of goods than younger vendors (Clark 1994). The first of the three scenarios is the most likely. The inverse relationship between education and participation in the bat-bushmeat trade, together with higher average age of those participating, could indicate that, as Ghanaians move into urban areas and stay in school longer, bat bushmeat use could start to decline.

We found a strong association between gender and roles in the bat-bushmeat commodity chain, with hunters primarily being male and vendors female, and this is consistent with the cultural norms of rural Ghanaian society (Clark 1994). If there is a difference in risk of disease from hunting and butchering bats, this therefore introduces a differential risk between the sexes. Although no single cultural or ethnic group dominated bat-bushmeat activities, there was a particularly high rate of hunting activity in the Volta region which could mark Volta markets as potential foci for future management, from education to sustainable hunting programmes. Alternatively, bat-bushmeat hunting—and the corresponding at-risk groups—could continue to become focussed in more rural areas.

There were notable discrepancies between stated perceptions and reported behaviours. Over half of respondents thought participation in bat bushmeat related to a particular tribe, yet few consumers identified bats as a traditional food. While the lack of personal identification of culture as a rationale for bat-bushmeat consumption could simply reflect the unconscious nature of cultural influence, the high rate of consumption among people in the Volta region and the lower prevalence among those ancestrally from Volta but living in Accra indicates that location has a stronger influence than ethnicity.

There is an on-going debate in the literature about whether bushmeat is a survival food or an economic luxury (Davies and Brown 2007), likely because patterns of use vary both spatially and temporally. In Ghana, bat bushmeat seems to have both subsistence and luxury functions; the large numbers of hunters who hunt for themselves or who keep some of their catch suggests that bats provide a readily available source of animal protein. At the same time, high taste ratings among consumers and relatively high prices charged (Kamins et al. 2011) suggest that bat meat is preferred by a number of people in Ghana. Understanding the drivers of bat-bushmeat consumption and then utilising that information to tailor interventions can increase the efficacy and acceptability of such interventions.

Multiple factors are at play in the perception of disease risk and participation in bat-bushmeat activities. Generally, the risk of bat bushmeat was considered to be greatest by those who did not consume the meat and lowest by those who hunted or sold the bats. It is unclear whether a lack of familiarity with bats leads to a higher perception of risk, or if the perception of risk decreases the likelihood of involvement with bat bushmeat. Also, we found an association between decreased participation in bat-bushmeat activities and living in urban environments and schooling, possibly suggesting that continued urbanisation trends and improvements in education could reduce the use of bats as bushmeat. This influence could be countered, however, if increased household income leads to increased bushmeat consumption (Fa et al. 2009).

The focus groups offered more in-depth understanding of participants’ likely reactions to interventions regarding bat bushmeat. Laws and fines alone are unlikely to induce change. Our data, together with that of others (e.g., Cooney 2003), suggest that regulations by themselves are not effective solutions. While only some of our respondents would be willing to risk paying fines if they continued to earn enough from selling bat bushmeat, essentially no one knew of the existing hunting laws in Ghana, suggesting that enforcement is a major issue. Additionally, possible health risks appeared to be more of a deterrent than fines. Some focus group respondents suggested, unprompted and before the education piece, that disease risk could motivate them to stop, yet perception of risk was only weakly related to non-participation in bat-bushmeat activities. This contrast is consistent with the statement made by Wilkie (2006): people can readily perceive risk and even intellectually acknowledge desire to reduce that risk, but actual behaviour might not change.

If zoonotic pathogen spillover from bats were to occur in Ghana, the information obtained from this study will be invaluable in targeting specific groups at-risk and disease control measures. Prior to our studies, little was known about bat hunting for bushmeat in Ghana and our findings did not support local perceptions of who was involved. Elsewhere, authorities have responded to zoonotic outbreaks by targeting specific groups unfairly. During the global outbreak of H1N1 in 2009, for example, Egyptian officials used the origins of the “swine flu” virus to justify the slaughter of millions of pigs owned by the marginalised Christian community (Tadros 2010). Similarly, a Bangladeshi community condemned an unconventional imam and his followers as “punished by God” after they experienced an unusually high rate of Nipah disease (Blum et al. 2009). In the light of such occurrences, understanding both actual and perceived risk factors is vital. If a bat-borne zoonotic disease outbreak were to occur in Ghana, public health officials would have to take care that particular groups were not unnecessarily implicated—as should anyone proposing actions in any settings where such divisions are thought to exist. Further conflict could arise between the low perception of disease risk from bat bushmeat in Ghana and concurrent belief that bat bushmeat is a health food and the view that bats can cause disease in humans. Such conflicts would not be restricted to Ghana, but could extend to many countries in the world where various cultures may use bats and bat products for medicines, charms, potions and other health-related products (Allen 1939).

Thirdly, there may not be a simple way to minimise the risks of zoonotic spillover from bats. Bat hunting is a highly seasonal occupation (Kamins et al. 2011), which requires no input during the rest of the year and, like all bushmeat hunting, can be started and dropped at will. Conversely, rearing domestic animals—one possible sustainable solution for reducing bushmeat hunting—requires continuous activity throughout the year on a daily basis. Although many programmes suggest economic opportunity as the major motivation behind livelihood choices and success of alternatives (Hilson and Banchirigah 2009), it may not be enough on its own. Integrating substantiated disease risk with economic opportunity could be effective, but the alternative may still have to be more attractive in other ways than bat hunting. Feelings of external benefit or responsibility are also inherent dangers of external approaches (Bowen-Jones et al. 2002). We need more cultivation of grassroots demand for alternative livelihoods and greater effort to identify factors that limit developing local ownership (Pollnac et al. 2001).

Understanding the complexities of the socio-economic environment in which zoonotic outbreaks occur is vital for ensuring preventative and control measures are correctly informed. The current study forms part of the evolution of emerging disease management, in which disciplines from sociology, political science and management science to virology, microbiology and epidemiology join forces to effectively and justly manage disease events.

## Appendix 1: Main Questionnaire

- Do you hunt bats? [yes, no]
  - How often do you hunt bats? [daily, 2–5 times a week, weekly, monthly, less often]
  - How many bats do you catch each time you hunt? [1–2,2–4,5–10, 10–20, more than 20]
  - (If hunting right now) How many bats did you catch today? [number]
  - How many bats did you catch last time you hunted them? [number]
  - Where do you hunt your bats? [location]
  - What method or methods do you use to catch/kill a bat? [catapulting, gun, snare, poison, scavenge, multiple, other]
- Do you sell them or keep them?
  - Do you keep any of your captured bats for yourself? [yes, no]
    (i) If so, how many did you keep the last time you kept some bats? [number]
  - How often do you sell bats? [daily, 2–5 times a week, weekly, monthly, less often]
  - How much of your income do you get from hunting bats? [very little, some, half, most, all]
  - About how much is that proportion?
  - How does the time of year affect your income from bats? [open]
  - How much did you sell your bats for last time you sold them? [GHC]
  - Has the price of bats gone up, down or stayed the same?
  - Is this because there are more/fewer bats or because more/fewer people want them?
  - How far do you have to travel between catching a bat and selling it? [km]
    (i) how is the meat transported/care for? [open]
  - To whom do you sell your bats? [see if I can meet them]
  - Do you hunt other animals as well? [yes/no] How about “grasscutter” “duiker”
  - Do you do anything else for money?
- Do you sell bats? [yes/no]
  - How often do you sell bats? [daily, 2-5 times a week, weekly, monthly, less often]
  - How much do you sell a bat for? [GHC]
  - How much did it cost for you to buy the last bats you got? [GHC]
  - How many bats did you get today? [number]
    (i) Yesterday? [number]
    (ii) On average? [number]
  - How many bats did you sell today? [number]
    (i) Yesterday? [number]
    (ii) On average? [number]
  - How much of your income do you get from selling bats? [very little, some, half, most, all]
  - How do you get your bat meat? [killed but unprepared, freshly prepared, smoked, other]
  - Where do your bats come from (geographically)? [location]
  - How does the time of year affect your income from bats? [open]
  - Has the price of bats gone up, down or stayed the same?
  - Is the because there are more/fewer bats or because more/fewer people want them?
  - To whom do you sell your bats?
  - From whom do you get your bats?
- Do you eat bats? [yes/no]
  - How often do you eat bat meat? [daily, 2–5 times a week, weekly, monthly, less often]
  - Why do you eat bat meat? [open, but (e) (g) because its available, cheap, tasty, medicinal]
  - How is the bat meat prepared [fresh, already butchered, smoked, or fully prepared]?
  - How much do you pay for a smoked bat?
  - How much do you pay for bat soup?
  - How does the time of year affect your consumption of bats? [open]
- Do you prepare/butcher bats? [yes/no]
  - How often do you prepare bats yourself ((i) (e) butcher or smoke them)? [rarely, sometimes, often, always]
  - What do you do? [open, eg remove guts, skin, chop up, smoke]
- Do you think just hunting bats might make you sick? [yes/no]
- Do you know anyone who has gotten sick from hunting bats? [yes/no]
- How likely do you think it is that someone would get sick? [very unlikely, unlikely, somewhat likely, likely, very likely]
- Do you think these activities pose a [a small threat, a significant threat, a serious threat]?
- Do you think that just butchering or preparing bats can make someone sick? [yes/no]
- Do you know anyone who has gotten sick from butchering or preparing bats? [yes/no]
- How likely do you think it is that someone would get sick? [very unlikely, unlikely, somewhat likely, likely, very likely]
- Do you think these activities pose a [a small threat, a significant threat, a serious threat]
- Do you think that just eating bats can make someone sick? [yes/no]
- Do you know anyone who has gotten sick from eating bats? [yes/no]
- How likely do you think it is that someone would get sick? [very unlikely, unlikely, somewhat likely, likely, very likely]
- Do you think these activities pose a small threat, a significant threat, or a serious threat?
- Do you feel that people want bat meat more than, less than or the same as they did five years ago?
- Who do you think most commonly eats bats?
- Which do you prefer, bush meat or domestic meat?
- Do you ever trade bats for something other than money, or use something other than money to trade for a bat? [yes/no]
  (i) What did you trade?
- Can you put these cards in order of which you like to eat best to which you like to eat least? [grasscutter, bat, pork, chicken, beef, goat, duiker]
- Can you put these cards in order of which you eat most often to which you eat least often? [grasscutter, bat, pork, chicken, beef, goat, duiker]

Demographic:

Sex

Age

Education

Group

From

Lived

## Appendix 2: Pre-test and Post-test (same questions)

Hi, my name is Alexandra and this is John. We are from the University of Ghana in Legon, doing a project on bats as bushmeat. We wanted to ask you a few questions, to understand how you think about bats. You don’t have to answer any questions, and we can stop whenever you want. I can’t pay you, but your answers will help us understand how people and bats interact.

I’m going to ask you some questions, then talk with you a little bit about bats, and then ask you the same questions again to see if anything has changed.

## Appendix 3: Education Intervention Details

Education program 1: Zoonoses

1. You can get several different things from bats. A few examples are:
  - Nipah virus:
    (i) Confusion, unconsciousness, fever, headaches, difficulty breathing
  - A cousin of rabies:
    (i) Pain and numbness in limbs, inability to drink water, headache, excitability
2. How to prevent it
  - Don’t drink raw palm sap
  - Don’t eat fruit that a bat has eaten
  - Cover food and drink when underneath bats
  - Wash your hands after touching a bat (hunting or butchering it)
  - Wash the bite very well, and go to the doctor if you get bitten by a bat
3. It is not very common to get these diseases from bats. In fact, people have only become ill from Nipah in Asia. But bats in Ghana carry this disease, so we are studying whether or not it is possible for people to get sick from bats. However, there is no cure for these diseases, so we think it is better to be safe even if there is no risk than to get sick.

Education program 2: Conservation

1. Good things bats do for the environment
  - Because bats can fly, they can carry plant seeds to places where all the trees have been cut down. This helps forests grow back.
  - Some seeds grow better after they have been eaten and passed through a bat. This helps new plants grow.
  - Pollinate *Azadirachta indica* (neem tree) and endangered Baobob trees
2. Good things bats do for people
  - Bats pollinate important trees for people, like hardwoods (e.g. teak)
  - Bats help *Ficus* (fig) and *Psidium* (guava) trees grow
3. How quickly bats reproduce—game with marbles
4. What will happen to people if the bats are gone
  - Trees will grow back slower, or not at all
  - Cut areas will be slow to come back
  - There will be no bats to hunt
  - People who need wood or fruits that bats help will not have those products
